# Association of Cytotoxic T-Lymphocyte Antigen-4 (*CTLA-4*) Genetic Variants with Risk and Outcome of Cutaneous Melanoma

**DOI:** 10.3390/ijms252212327

**Published:** 2024-11-17

**Authors:** Ana Maria Castro Ferreira, Juliana Carron, Gabriela Vilas Bôas Gomez, Vinicius de Lima Vazquez, Sergio Vicente Serrano, Gustavo Jacob Lourenço, Carmen Silvia Passos Lima

**Affiliations:** 1 Laboratory of Cancer Genetics, School of Medical Sciences, State University of Campinas, Campinas 13083-970, SP, Brazil; castroferreiraana@gmail.com (A.M.C.F.); jcarron@unicamp.br (J.C.); gabivbg@gmail.com (G.V.B.G.); guslour@unicamp.br (G.J.L.); 2 Department of Anesthesiology, Oncology and Radiology, School of Medical Sciences, State University of Campinas, Campinas 13083-970, SP, Brazil; 3 Melanoma and Sarcoma Surgery Department, Barretos Cancer Hospital, Barretos 14784-400, SP, Brazil; viniciusvazquez@gmail.com; 4 Department of Medical Oncology, Barretos Cancer Hospital, Barretos 14784-400, SP, Brazil; svserrano@hotmail.com

**Keywords:** cutaneous melanoma, *CTLA-4*, single nucleotide variants, risk, prognosis

## Abstract

This study aimed to verify whether germline single nucleotide variants (SNV) in *CTLA-4* gene, c.-1765C>T, c.-1661A>G, c.-1577G>A, and c.-1478G>A, influence the risk, clinicopathological aspects, and survival of patients with CM, as well as its functional consequences. A total of 432 patients with CM and 504 controls were evaluated. *CTLA-4* genotypes were identified by real-time polymerase chain reaction (RT-PCR) and expression of *CTLA-4* by quantitative PCR (qPCR) and luciferase assay. Cell cycle, proliferation, apoptosis/necrosis, and migration analyses were performed in SK-MEL-28 and A-375 cell lines modified to present homozygous ancestral or variant genotypes by CRISPR technique. Individuals with the *CTLA-4* c.-1577 AA genotype and the combined *CTLA-4* c.-1577 and c.-1478 AA + AA genotypes were at 1.60- and 3.12-fold higher risk of developing CM, respectively. The *CTLA-4* c.-1577 AA genotype was seen as an independent predictor of worse event-free survival and was also associated with higher gene expression, higher cell proliferation, lower cell apoptosis, and higher cell migration. Our data present, for the first time, evidence that *CTLA-4* c.-1577G>A alters the risk and clinical aspects of CM treated with conventional procedures and may be used for selecting individuals for tumor prevention and patients for distinct treatment.

## 1. Introduction

Cutaneous melanoma (CM) is the most aggressive form of skin cancer and is responsible for most skin cancer-related deaths [1]. While melanin is protective against UV radiation, its secretion by CM cells can promote tumor progression and metastasis by modulating the tumor microenvironment [1]. Recent studies conducted by our group focused on the influence of single nucleotide variants (SNV) on immune-related genes in melanoma and identified associations between SNVs in the *PDCD1* [2] and *TNFRS1B* [3] genes with CM risk and/or outcomes. Understanding these genetic factors may contribute to improved prevention, early diagnosis, and development of targeted therapies for CM.

**Figure 1:**
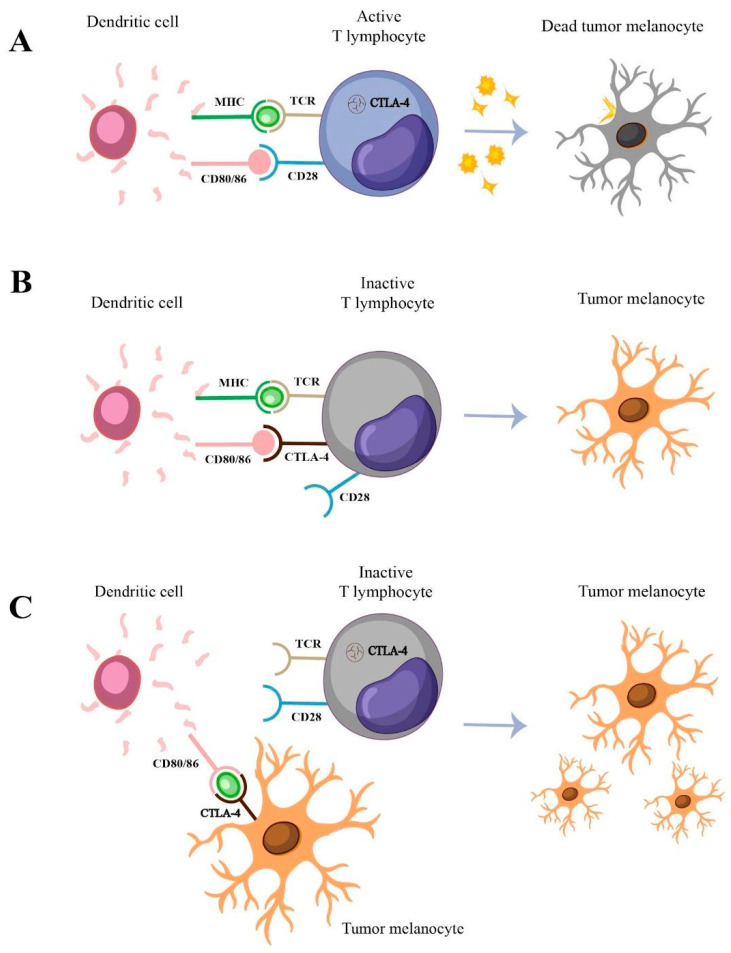
Signaling pathway of CTLA-4. The dendritic cell identifies antigens in the microenvironment and, with the help of the major histocompatibility complex (MHC), presents these antigens to inactive T lymphocytes, initiating the activation process (**A**). The first step occurs when the MHC binds to the T-cell receptor (TCR). After this binding, surface proteins of the CD80/86 family on dendritic cells bind to the CD28 receptor on T lymphocytes, promoting increased cell proliferation, enhancing cytokine production, and combating tumor melanocytes. During activation, CTLA-4, initially stored in vesicles within the cytoplasm, is released, becomes a receptor, and binds with higher affinity than CD28 to the CD80/86 family proteins (**B**). This binding leads to the inactivation and apoptosis of T lymphocytes, allowing tumor melanocytes to survive, as lymphocytes do not target them, blocking T-lymphocyte activation from the binding of melanocytes to antigen-presenting cells (**C**). By binding to tumor melanocytes, the dendritic cell prevents the antigen from being presented to T lymphocytes, thus inhibiting T-lymphocyte activation. As a result, the tumor melanocyte evades the immune system’s response.

Cytotoxic T-lymphocyte antigen-4 (CTLA-4) is an immune checkpoint molecule expressed primarily on activated T cells and regulatory T (Treg) cells [4]. Its interaction with the B7 ligands (CD80/CD86), expressed on antigen-presenting cells (APC), inhibits cell proliferation, cytokine (IL-2, IFN-γ) production, and cell cycle progression, inhibiting T-cell activation [4]. Maintained high exposure to antigens in the tumor microenvironment induces a state of dysfunction in anti-tumor effector T cells, called T-cell exhaustion, causing failure in cancer cell elimination as a consequence (Figure 1A,B) [5]. CTLA-4 is also known to be expressed in different types of non-T cells, such as monocytes [6] and CM cells [7]. In tumor cells, the presence of checkpoint molecules can contribute to an escape mechanism against the immune system’s action, since T-cell activation is inhibited and favors tumor expansion and proliferation (Figure 1C) [8].

As CTLA-4 is encoded by the *CTLA-4* polymorphic gene, SNVs can influence protein function [4]. Located in the *CTLA-4* promoter region, c.-1765C>T (rs11571315) [9], c.-1661A>G (rs4553808) [10], c.-1577G>A (rs11571316) [11], and c-1478G>A (rs62182595) [12] SNVs may contribute different roles in this region. *CTLA-4* c.-1765C>T and c.-1478G>A were associated with a higher risk of developing red blood cell transfusion-associated adverse reactions due to the inhibition of regulatory mechanisms of lymphocyte activity, mainly from Treg cells [12], and the GG genotype of *CTLA-4* c.-1661A>G and c.-1577G>A was associated with a better response to the blocker ipilimumab and long-term survival in metastatic CM patients [13], but, to our knowledge, the roles of *CTLA-4* SNVs in CM are still unknown.

The present study aimed to verify, for the first time, associations of *CTLA-4* c.-1577G>A, c.-1478G>A, c.-1765C>T, and c.-1661A>G SNVs with the risk and clinicopathologic aspects of CM and prognosis of patients treated with surgery and conventional chemotherapy, as well as to conduct functional analyses for clarifying their biological consequences.

## 2. Results

### 2.1. Clinicopathological and Tumor Aspects

Clinicopathological aspects of patients are presented in Table 1. The median age of patients was 54 years, and a discreet predominance of women was observed in the patient group. Most patients had white skin color, phototype I–III, brown or black eyes, and were exposed to the sun. Patients were older, were more exposed to the sun, and had white skin, phototype I–III, and blue or green eyes more frequently than the controls; thus, all analyses involving patients and controls were adjusted for differences in age, skin color, phototype, eye color, and sun exposure found in patients and controls. We observed that around half the number of patients had tumors in the trunk, with a superficial histological type, IV or V Clark level, and at early clinical stage (stage I).

### 2.2. Genetic Variants in the Risk of CM

Controls were in Hardy–Weinberg equilibrium (HWE) for the *CTLA-4* c.-1765C>T (X^2^ = 0.29, *p* = 0.59), c.-1661A>G (X^2^ = 4.15, *p* = 0.40), c.-1577G>A (X^2^ = 0.17, *p* = 0.68), and c.-1478G>A (X^2^ = 2.49, *p* = 0.11) loci. Patients were in HWE for the *CTLA-4* c.-1765C>T (X^2^ = 1.48, *p* = 0.22) and c.-1661A>G (X^2^ = 0.57, *p* = 0.45) loci but nor for the c.-1478G>A (X^2^ = 6.41, *p* = 0.01) and c.-1577G>A (X^2^ = 16.09, *p* = 0.0001) loci, showing a preferential distribution for one genotype. CAAG, TGAG, and CGAG haplotypes of the SNVs were considered for the study.

The *CTLA-4* c.-1577 AA genotype was more common in patients than in controls; individuals with this genotype were at a 1.60 times greater risk of presenting CM than individuals with the GG or GA genotypes. An excess of the combined genotype was seen in patients than in controls; individuals with the combined genotype had a 3.12 times greater risk of presenting CM than those with the remaining genotypes, as shown in Table 2. Similar frequencies of CAAG, TGAG, and CGAG haplotypes were seen in patients and controls, showing similar risks for disease in carriers and non-carriers of the haplotypes presented in Supplementary Table S1.

### 2.3. Genetic Variants with Clinicopathologic Aspects

The *CTLA-4* c.-1765 CC or CT genotype was more common in patients aged less than or equal to 54 years than in older patients, and an excess of the c.-1661 AA genotype was seen in males compared to females, as shown in Table 3. Similar frequencies of the combined genotypes of the *CTLA-4* variants were seen in patients stratified by clinical aspects (Supplementary Table S2).

Similar frequencies of isolated or combined genotypes of the *CTLA-4* variants were seen in patients stratified by tumor location, histological type, Clark level, and stage (Supplementary Table S3).

### 2.4. Survival Analysis

Survival was assessed in 411 out of the 432 patients included in the study; twenty-one patients were excluded from the analysis due to a lack of reliable survival data. The survival analysis was not compromised by the loss of follow-up for these patients, as the characteristics of the total CM group and the patients included in the analysis did not differ. The median follow-up time assessed was 60 months (range: 5–423 months). As to the last survival analysis (14 May 2024), 130 patients had died (50 from the disease effects and 80 from other causes), and 281 patients were alive (16 with the disease and 265 without the disease). Eighty-eight patients experienced a tumor relapse.

At 60 months of follow-up, event-free survival (EFS) was lower in patients aged over 54 years (65.4% vs. 76.6%, *p* = 0.002), with non-superficial tumor (63.7% vs. 92.6%, *p* < 0.0001), Clark levels III–V (35.8% vs. 88.4%, *p* < 0.0001), and at stages III or IV (68.1% vs. 83.5%, *p* < 0.0001), and in patients with *CTLA-4* c.-1577AA genotype (52.2% vs. 71.1%, *p* = 0.02) than in the other patients (Kaplan–Meier estimates) (Supplementary Figure S2). All these factors were predictors of patients’ EFS in univariate Cox analysis. Patients over 54 years of age with non-superficial tumor, Clark level III–IV, stage III or IV, and with *CTLA-4* c.-1577 AA genotype had a 1.80-, 4.87-, 4.37-, 3.43-, and 1.60-times greater chance of recurrence, disease progression, or death related to tumor effects, respectively. In Cox multivariate analysis, patients over 54 years of age, histological type, Clark level, stage, and *CTLA-4* c.-1577G>A genotypes were independent predictors of patients’ EFS. Patients over 54 years of age with non-superficial tumor, tumors of Clark level III–V, stage III–V, and with *CTLA-4* c.-1577AA genotype had 1.75-, 3.46-, 3.00-, 2.39-, and 1.75-times greater chance of presenting disease progression than others, respectively, as shown in Table 4.

At 60 months of follow-up, melanoma-specific survival (MSS) was lower in patients older than 54 years (56.7% vs. 83.3%, *p* = 0.0002), with tumor of Clark level III–V (44.0% vs. 66.3%, *p* < 0.0001), stage III or IV (49.2% vs. 96.3%, *p* < 0.0001), and AA genotype of the SNV c.-1577G>A (33.9% vs. 66.5%, *p* = 0.04) than in other patients (Kaplan–Meier estimates), as shown in Supplementary Figure S3. In univariate Cox analysis, all these factors were predictors of MSS; patients older than 54 years, with non-superficial tumors, tumors of Clark level III–V, stage III or IV, and *CTLA-4* c.-1577 AA genotype had 2.33-, 5.56-, 5.94-, 3.78-, and 1.60-times more chances of dying by tumor relapse, progression, or effects than others, respectively. In multivariate Cox analysis, age, tumor histological type, Clark level, and stage were independent predictors of MSS. Patients over 54 years of age, with non-superficial tumor, tumor of Clark level III–V, and III or IV stage had 2.44-, 3.84-, 4.19-, and 3.06-times greater chances of progression to death by tumor effects than remaining patients, respectively, as shown in Table 4.

Similar EFS and MSS were seen in patients stratified by *CTLA-4* combined genotypes, as presented in Supplementary Table S4.

Based on the results of risk analyses, clinical aspects of patients, biological aspects of the tumor, and survival of CM patients, we selected *CTLA-4* c.-1577G>A as the SNV of greatest interest for this study, thus making it the object of functional analyses.

### 2.5. CTLA-4 Protein in SK-MEL-28 and A-375 Cell Lines

CTLA-4 protein was identified in the cytoplasm of SK-MEL-28 and A-375 cells but not in the cell membranes. As this was a qualitative analysis, no numerical values were assigned to the labels (Supplementary Figure S4).

### 2.6. CTLA-4 Expression

**Figure 2:**
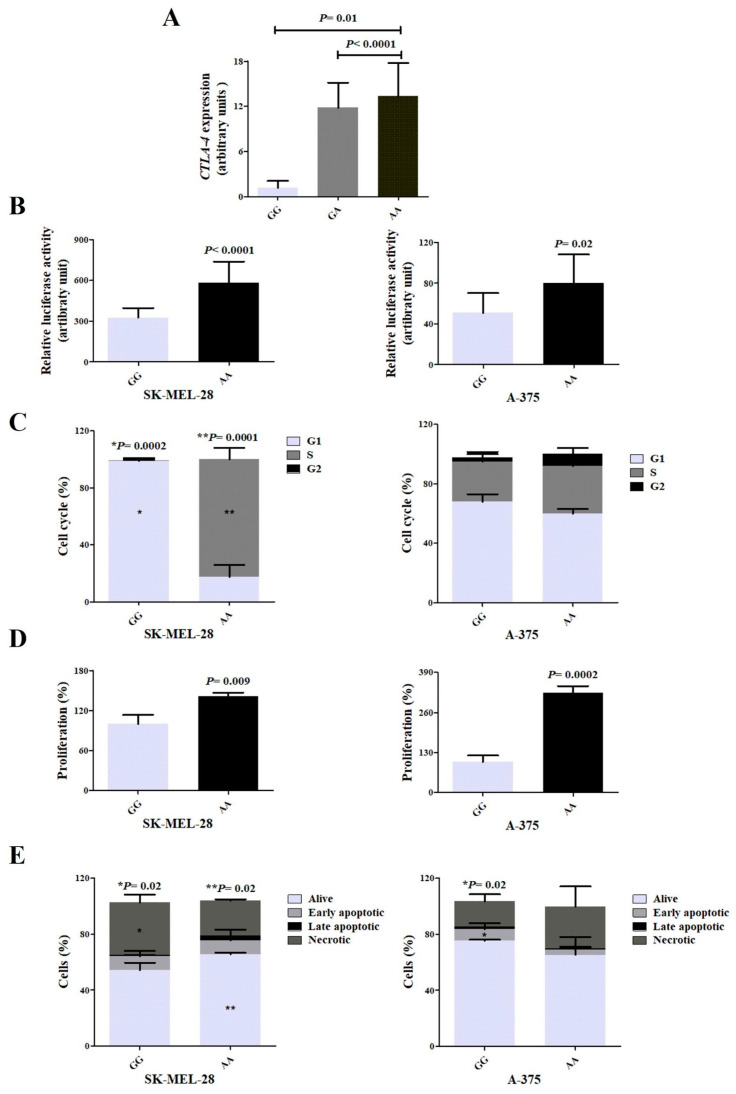
Functional analyses of the ancestral and variant genotypes of the *CTLA-4* c.-1577G>A. Analysis of *CTLA-4* gene expression in peripheral blood of patients with cutaneous melanoma (**A**). Gene expression was higher in patients with AA genotype than in those with GG genotype. Relative luciferase activity in SK-MEL-28 and A-375 melanoma cell lines transfected with the ancestral plasmid (GG genotype) or with the variant plasmid (AA genotype) (**B**). Luciferase activity was higher in cells with AA genotype than in cells with GG genotype. Assessment of the cell cycle in strains modified to present ancestral and variant genotypes (**C**). Cells were identified in the G1, S, and G2 phases using flow cytometry. A higher percentage of SK-MEL-28 cells with the GG genotype was found in the G1 phase compared to those with the AA genotype *, and a higher percentage of SK-MEL-28 cells with the AA genotype was found in S phase compared to those with GG genotype **; a similar percentage of A-375 cells were seen in the G1, S, and G2 phases. Cell proliferation in SK-MEL-28 and A-375 melanoma cell lines (**D**). A higher percentage of SK-MEL-28 and A-375 cells with AA genotype was found in proliferation when compared to those with the GG genotype. Analysis of the assessment of apoptosis and necrosis by flow cytometry with stimulation of the immunotherapy drug ipilimumab (**E**). A higher percentage of SK-MEL-28 cells with GG genotype was found in necrosis compared to those with the AA genotype *; a higher percentage of cells with the AA genotype were alive compared to those with the GG genotype **; the A-375 cells with the GG genotype were in initial apoptosis when compared to cells with the AA genotype.

*CTLA-4* gene expression was higher in patients with the AA genotype of the *CTLA-4* c.-1577G>A than in patients with the other genotypes (8.31 arbitrary units (AU) ± 6.93 vs. 3.79 AU ± 4.94, *p* = 0.01) (Figure 2A).

### 2.7. Luciferase Activity

Relative luciferase activity was higher in SK-MEL-28 and A-375 cells transfected with the variant plasmid (AA genotype) than in cells transfected with the ancestral plasmid (GG genotype) of the *CTLA-4* c.-1577G>A (583.9 AU vs. 326.5 AU, *p* < 0.0001; 79.7 AU vs. 50.7 AU, *p* = 0.02, respectively) (Figure 2B).

### 2.8. Transcription Factor Binding

**Figure 3:**
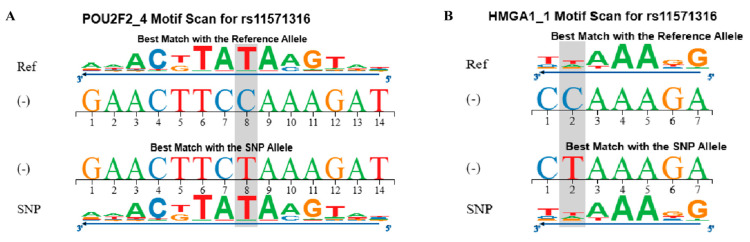
Transcription factor binding sites for the *CTLA-4* 1577G>A (rs11571316) single-nucleotide polymorphism (SNP). Binding of the transcription factor POUPF2 in the 3′-5′ direction of the *CTLA-4* gene (**A**). Binding of the transcription factor HMGA1 in the 3′-5′ direction of the *CTLA-4* gene (**B**). The gray square represents the SNP alleles.

The analysis indicated that the *CTLA-4* c.-1577A allele increases the binding affinity of POUP2F2 (Figure 3A) and HMGA1 (Figure 3B), resulting in a decrease in log odds from −22.4 to −16.4 (impact *p*-value = 0.002) and from −27.6 to −5.3 (impact *p*-value = 0.01), respectively.

### 2.9. Cell Cycle and Proliferation

A lower percentage of SK-MEL-28 cells with the GG genotype of the *CTLA-4* c.-1577G>A in the G1 phase was found compared with cells with the AA genotype (17.7% vs. 99.0%, *p* = 0.0002), and a higher percentage of SK-MEL-28 cells with the AA genotype was found in the S phase compared with cells with GG genotype (82.2% vs. 0.16%, *p* = 0.0001). Similar distributions of A-375 cells with GG and AA genotype of the *CTLA-4* c.-1577G>A were seen across cell cycle phases (Figure 2C). Proliferation was higher in SK-MEL-28 and A-375 cells with the AA genotype than in respective cells with the GG genotype (140.8% vs. 100%, *p* = 0.009; 322.6% vs. 100%, *p* = 0.0002, respectively) (Figure 2D).

### 2.10. Cell Apoptosis and Necrosis

A higher percentage of SK-MEL-28 cells with the AA genotype of the *CTLA-4* c.-1577G>A SNV were alive (65.7% vs. 54.2%, *p* = 0.02), and a lower percentage of cells with the AA genotype had undergone necrosis (25.2% vs. 37.5%, *p* = 0.02) in comparison with cells with the GG genotype. No differences were found in the percentages of cells undergoing apoptosis (early and late phases) in SK-MEL-28 cells with the AA and GG genotypes.

A lower percentage of A-375 cells with the AA genotype were in initial apoptosis than cells with the GG genotype (5.2% vs. 8.3%, *p* = 0.02). Similar percentages of living cells and cells in late apoptosis or necrosis were seen in cells with the AA and GG genotypes of the *CTLA-4* c.-1577G>A SNV (Figure 2E).

### 2.11. Cell Migration

**Figure 4:**
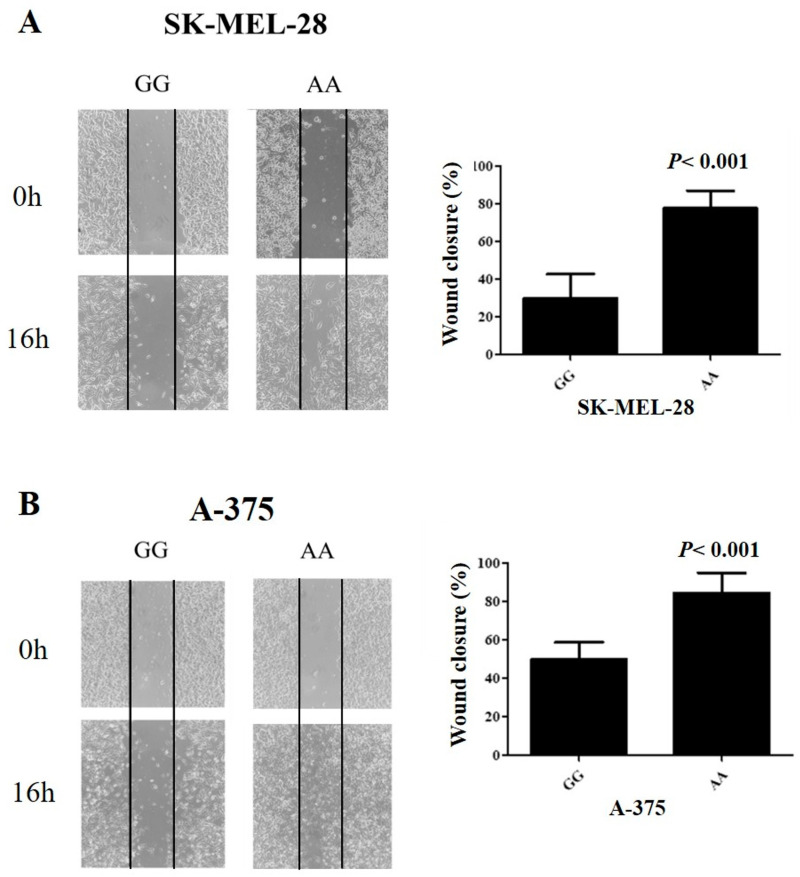
Cell migration by wound healing assay in melanoma cell lines SK-MEL-28 (**A**) and A-375 (**B**). Cells with the AA genotype of the *CTLA-4* c.-1577G>A single nucleotide variant showed a higher percentage of wound closure after 16 h.

An increased wound closure was seen with SK-MEL-28 and A-375 cells with the AA genotype of the *CTLA-4* c.-1577G>A compared with wound closure determined by cells with the GG genotype (84.8% vs. 15.2%, *p* < 0.001; 75.4% vs. 24.6%, *p* < 0.001, respectively) (Figure 4).

## 3. Discussion

This study evaluated the roles of the *CTLA-4* SNVs, c.-1765C>T, c.-1661A>G, c.- 1577G>A, and c.-1478G>A in the risk of CM, clinicopathological aspects, and survival of patients with CM. In addition, the c.-1765C>T SNV was considered of great interest and was evaluated by complementary functional studies.

Firstly, we found that the clinical aspects of our patients and pathological aspects of the tumor were like those seen in patients analyzed in other parts of Brazil [14] and worldwide [15]. Therefore, the sample included in the study was representative of CM and could be used to evaluate factors associated with the clinicopathological aspects of CM and the prognosis of patients.

Secondly, we found that the AA genotype of *CTLA-4* c.-1577G>A was associated with a 1.60-times higher risk of developing CM compared to individuals with the remaining genotypes. We also observed that individuals carrying the combined *CTLA-4* c.-1577AA and c.-1478AA genotypes were at a 3.12-times increased risk of developing CM compared to individuals with the remaining genotypes. Palacios et al. (2008) [16] identified higher expression of *CTLA-4* in patients with multiple sclerosis and the AA genotype of the c.-1577G>A SNV and association of the *CTLA-4* c.-1577AA genotype, and increased risk of acute lymphoblastic leukemia was also observed by Aref et al. (2023) [17]. We believe that individuals with the *CTLA-4* c.-1577 AA genotype of our population could be under a high *CTLA-4* mRNA level [16], which may have led to increased *CTLA-4* expression, greater downregulation of the immune system, and CM development as consequences [7].

Third, it was found that CC and CT genotypes of the *CTLA-4* c.-1765C>T SNV were more common in patients under 54 years of age, suggesting that they predispose individuals to early occurrence of the tumor. This may be associated with aspects observed in tumors with altered genetic components, such as those of hereditary origin syndromes which are, respectively, determined by high- and low-penetrance variants [18]. The AA genotype of *CTLA-4* c.-1661A>G was more frequent in men than in women. The higher frequency of the genotype in men may be related to underlying biological differences between the sexes, as observed with the presence of suppressor genes on the X chromosome, such as PPP2R3B, which encodes the PR70 protein, and it interferes with DNA replication and cell cycle progression, delaying the growth of CM in females and contributing to these disparities [19]. SNVs in distinct apoptosis-related genes were also found as plausible explanations for differences in the risk of CM in men and women [20].

Fourth, lower EFS was observed in patients with the *CTLA-4* c.-1577AA genotype. Carriers of this genotype had 1.75-times more chance of disease relapse or progression compared to patients with the remaining genotypes. The GG genotype of *CTLA-4* c.-1661A>G and c.-1577G>A were associated with a better response to the blocker ipilimumab and long-term survival in metastatic CM patients [13]. The *CTLA-4* c.-1661 GG genotype was not associated with patient survival in our study, and differences in results found in our study and a study conducted by Queirolo et al. may be attributed to distinct sample sizes (411 patients in our study and 173 patients in Queirolo et al.’s study), distinct primary melanoma subtype (only CM in the current study and CM, mucosal, ocular, and melanoma of unknown primary tumor site in Queirolo et al.’s study), distinct tumor stage (all stages in our study and stage IV in Queirolo et al.’s study), and distinct treatment (conventional procedures and chemotherapy in our study and ipilimumab in Queirolo et al.’s study) [13]. Therefore, it is inferred that patients with the AA genotype of the *CTLA-4* c.-1577G>A SNV present a significant increase in *CTLA-4* mRNA level and, consequently, an increased expression of *CTLA-4* on the cell surface [16]. Thus, it would be reasonable to hypothesize that lower *CTLA-4* expression level would result in the downregulation of effector T cells and reduced interaction with B7 ligands, potentially facilitating the blocking ability of ipilimumab, ensuring better survival for carrier individuals of the GG genotype compared to patients with the AA genotype [13,16]. At this point of the discussion, it is worth commenting/postulating that the *CTLA-4* c.-1577G>A SNV can alter the survival of CM patients treated only with surgical tumor resection and conventional chemotherapy, as seen in our cases, possibly due to deregulation of the immune system.

We observed higher expression and higher luciferase activity of the *CTLA-4* gene in carriers of the homozygous variant AA genotype of SNV c.-1577G>A than in carriers of other genotypes, which corroborates previous studies [7,13]. The greater luciferase activity observed in SK-MEL-28 and A-375 cells is associated with a possible influence on the promoter region of the *CTLA-4* gene and transcriptional activity [17].

In silico analysis indicated that POU2F2, a transcription factor in the POU family, plays significant roles in cancer progression and development, as well as in melanocyte development and CM progression, as it responds to MAPK pathway activation and modulates melanocyte-inducing transcription factor (MITF) levels, suppressing the differentiated melanocytic phenotype and enhancing tumor metastasis [21]. Overexpressed in several aggressive cancers, HMGA1 functions as a master regulator of gene expression, influencing cellular processes such as transcription, cell cycle progression, differentiation, DNA repair, and neoplastic transformation [22]. Furthermore, it still plays a crucial role in cancer inflammation by promoting macrophage recruitment and activating NF-κB-CCL signaling, contributing to tumor growth [22]. The HMG family forms a complex with CXCL12 that binds to the CXCR4 receptor, enhancing cell migration [23]. The non-canonical NF-κB p52/RelB pathway is essential to sustain a CXCL12 autocrine loop in cells that migrate in response to HMG [24]. These findings suggest that POUP2F2 and HMGA1 transcription factors play a crucial role in melanoma progression, proliferation, and migration and, associated with an increase in the binding site related to the A allele, corroborate the results obtained in our study.

A higher percentage of SK-MEL-28 cells with the AA genotype in the S phase indicates a higher cell proliferation seen in our study, since cell proliferation is a fundamental process involving cell growth and division, crucial for tumor development, as well as the S phase of the cell cycle [25].

A higher percentage of SK-MEL-28 cells with the AA genotype as living cells and a high percentage of cells with the GG genotype in necrosis were seen in our study. These results are like those found in patients by Queirolo et al. (2017), in which the AA genotype showed a worse response to ipilimumab compared to the GG genotype. The effect of immunotherapy on cell analysis was also found in the A-375 cell line in our study, in which a lower percentage of cells with the AA genotype underwent apoptosis at an early stage in comparison with cells with the GG genotype [13].

Cells that can escape apoptosis, migrate from the initial site, and grow elsewhere define the malignant nature of solid tumors [26]. SK-MEL-28 and A-375 cells with the AA genotype showed greater migration in a short time than cells with the GG genotype. *CTLA-4* signaling is known to increase cell motility and migration of specific chemokines, such as the CCL family and CXCL12, which, associated with the transcription factor HMGA1, increase the expression of the chemokine receptors CCR5 and CCR7. This migration-enhancing effect is mediated by PI3K-dependent Akt activation, leading to cytoskeletal rearrangements necessary for cell movement [27]. Therefore, cells with higher *CTLA-4* expression are expected to have greater migratory capacity, since there will be greater signaling of chemokine receptors expressed in melanocytes, which play crucial roles in melanoma progression and metastasis [28]. CCR5 is highly expressed in melanoma cells and is positively associated with tumor malignancy, increasing epithelial–mesenchymal transition and metastasis [29]. CCR7 expression in CM cells allows them to respond to CCL21, a chemokine produced by lymphatic endothelial cells [30]. This interaction facilitates the directed growth and migration of melanoma cells toward the lymph nodes, favoring metastasis [31]. The expression of CXC chemokines correlates with high clinical risk and poor prognosis in melanoma, becoming a potential marker of tumor aggressiveness, and also contributing to tumor growth and progression, which corroborates the results obtained in our study [30,31].

We are aware of the limitations of this study. The results of the association of genotypes with risk and clinical aspects of CM and survival of CM patients were obtained by analysis of patients from a single public oncology service, in which immunotherapy is not available; therefore, these results should be validated in a larger epidemiological study with CM patients and controls from ethnically diverse populations and in a cohort of patients treated with and without anti-CTLA-4 agents. In addition, blood donors are commonly used as controls in medical research, including genetic studies [32]. However, these individuals tend to be healthier than the general population, which could introduce a bias in our study.

## 4. Materials and Methods

### 4.1. Study Population

A total of 432 patients with CM were evaluated, and they were diagnosed and treated at the Clinical Oncology Service of the General Hospital of the State University of Campinas and the Cancer Hospital of Barretos of the Pio XII Foundation. Patients with acral melanoma were excluded from the analysis because they had distinct phenotypic and genetic features, suggesting biological differences from those with other CM. The control group consisted of 504 blood donors who attended the Hematology and Hemotherapy Center of the same university during the same period.

### 4.2. Clinical Pathological Aspects, Tumor Aspects, and Treatment

Patients and controls completed standardized questionnaires to provide clinical information, such as age, sex, and sun exposure. Regarding sun exposure, patients were classified as sun-exposed when exposed to more than two hours of sunlight per day for more than ten years and not exposed to the sun when exposed to fewer hours and/or a shorter time interval in years [33]. The phototypes were classified following Fitzpatrick’s classification [34]. Tumor aspects and survival data of patients were obtained from their medical records. Tumors were measured by Clark levels [35], and the clinical stage was defined by the 7th American Joint Committee on Cancer Criteria [36,37].

Patients with localized tumors underwent resection, and patients with clinically positive lymph nodes or histologically infiltrated tumors underwent lymphadenectomy. Patients with single-operable metastasis or recurrence were treated with surgical resection, while patients with inoperable recurrence or multiple metastases were treated with dacarbazine chemotherapy. Radiation therapy was used in patients with hemorrhagic lesions and bone or brain metastases. The patients included in this study did not use immunotherapies.

### 4.3. SNV Selection

All steps involved in selecting SNVs for the study are presented in Supplementary Figure S1. Firstly, all SNVs in the gene were identified using the Ensembl platform. A total of 784 SNVs were found and categorized by region into the 5′UTR region (*n* = 32), promoter region (*n* = 313), exon (*n* = 151), and 3′UTR region (*n* = 288). Subsequently, each SNV was researched in the literature using the National Center for Biotechnology Information (NCBI), looking for studies in which the SNVs had already been described or had been evaluated in CM. This left 2 SNVs in the exon, 6 in the promoter region, and 2 in the 3′UTR region.

The sample size of each SNV was calculated based on the International HapMap Project, and the minimum allele frequency (MAF) was calculated using the Variant Effect Predictor (VEP) [38]. After analysis, SNVs in the promoter region and with a MAF greater than 10% in the general population were included. In silico analyses were carried out using the SNP program to identify gene regions of interest, possible functional consequences of SNVs, and changes in transcription factor binding [39]. Finally, four SNVs were selected for this study, all located in the promoter region of *CTLA-4.*

### 4.4. Genotyping

Genomic DNA was obtained from the leukocytes of peripheral blood samples of patients and controls. Genotyping was performed by real-time polymerase chain reaction (RT-PCR) method using TaqMan^®^ SNV Genotyping Assay (Applied Biosystems^®^, Waltham, MA, USA) *CTLA-4* c.-1765C>T (rs11571315), c.-1661A>G (rs4553808), c.-1577G>A (rs11571316), and c.-1478G>A (rs62182595), following the manufacturer’s instructions. In all reactions, positive and negative controls were used. The 15% of genotype determinations were replicated in independent experiments with total concordance.

### 4.5. Survival Analysis of Patients with CM

The status of patients (alive with disease, alive without disease, death with disease, and death without disease) was obtained from medical records. EFS was calculated from the date of margin expansion to the date of first relapse, disease progression, death from disease, or last follow-up. MSS was calculated from the date of diagnosis to the date of death by melanoma or last follow-up.

### 4.6. Cell Culture

SK-MEL-28 (HTB-72™, American Type Culture Collection^®^, Manassas, VA, USA) and A-375 (CRL-1619™, American Type Culture Collection^®^) cell lines were cultured in Dulbecco’s Modified Eagle’s Medium (DMEM, Gibco, Waltham, MA, USA) supplemented with 10% fetal bovine serum (FBS, Gibco) and 100 U/mL penicillin-streptomycin (P/S, Gibco). All cells were cultured in a humidified atmosphere of 5% CO_2_ at 37 °C (Thermo Scientific, Waltham, MA, USA). SK-MEL-28 and A-375 cell lines were authenticated using short tandem repeat analysis, and all experiments were performed with mycoplasma-free cells.

### 4.7. CTLA-4 Expression in SK-MEL-28 and A-375 Cell Lines

To analyze CTLA-4 expression on the cell membrane, the samples were washed and resuspended in 1X PBS and labeled with CD152 antibody. To analyze the gene expression in the cytoplasm, the cells were impermeable by Cytofix™ fixation buffer (BD Biosciences, Franklin Lakes, NJ, USA) according to the manufacturer’s instructions. Subsequently, the cells were labeled again with CD152 antibody and analyzed by Cytoflex (Beckman Coulter, Brea, CA, USA).

### 4.8. CTLA-4 Expression in Peripheral Blood Samples

*CTLA-4* gene expression was assessed by quantitative PCR (qPCR) using RNA from peripheral blood leukocytes of 92 patients. The samples were initially lysed using the TRIzol reagent (Life Technologies, Carlsbad, CA, USA) according to the manufacturer’s instructions. The RNA samples were subjected to complementary DNA (cDNA) synthesis using reagents from the commercial Superscript III RT kit (Life Technologies, USA). *CTLA-4* gene expression was performed by SYBR Green PCR Master Mix reagents (Applied Biosystems, USA) and specific primers for *CTLA-4* gene (forward: 5′-CTCGAGGTCTGGCATTAGGAAGTG-3′, and reverse: 5′-AAGCTTGCCTTTTCTGACCTGC-3′) in triplicate per sample, and a negative control without a template was included in each plate. Gene expression was normalized by the expression of β-actin gene (*BAC*) (forward: 5′ AAGAGATGGCCACG-GCTGCT-3′ and reverse 5′-TCGCTCCAACCGACTGCTGT-3′), and the relative expression level was normalized applying the arithmetic formula 2^−ΔΔCt^ cycle threshold method [40]. Results were expressed in AUs.

### 4.9. Dual-Luciferase Reporter Assay

The SNV of interest was considered the one with the best association with CM risk, clinicopathological aspects, and/or survival of patients with CM. After obtaining the results, the selected SNV was *CTLA-4* c.-155G>A. For luciferase assay, the promoter region of *CTLA-4* c.1577_G (ancestral allele) and c.-1577_A (variant allele) of individuals with known *CTLA-4* GG and AA genotypes were amplified by PCR with Platinum™ Taq DNA Polymerase High Fidelity (Thermo Scientific, USA). Specific primers with restriction site for *XhoI* (Forward: 5′-TTCTCGAGGCCCTTTCTGACTTCC-3′; where the *XhoI* site is underlined) and *HindIII* (Reverse: 5′-CTCTAAGCTTGTCATCTCCTCCAG-3′; where the *HindIII* site is underlined) (Thermofisher, Waltham, MA, USA) were used. The fragments were cloned into the pGL-3 basic vector (Promega, Madison, WI, USA) containing the firefly luciferase gene driven by the CMV promoter, according to standard protocols. After these procedures, the plasmids were obtained and named pGL3_luciferase_G and pGL3_luciferase_A.

SK-MEL-28 and A-375 cell lines were transiently transfected with plasmids pGL3_luciferase_G (ancestral allele), pGL3_luciferase_A (variant allele), and Renilla Luciferase Control Reporter (pRL) (Promega, USA) using Lipofectamine 3000 (Invitrogen, Waltham, MA, USA) according to the manufacturer’s instructions. After 48 h from transfection, luciferase assays were performed with Dual Luciferase Assay Kit (Promega, USA) according to standard protocols. Tests were performed in triplicate and three independent experiments.

### 4.10. Transcription Factor Binding Motif Analysis

The predicted binding scores of transcription factors (TFs) were calculated using the atSNP program (http://atsnp.biostat.wisc.edu/) (accessed on 3 September 2024) [41]. This analysis aimed to assess whether the c.-1577G>A SNV affects the TF binding site in the promoter region of the *CTLA-4* gene. The evaluation focused on the significance of changes in TF binding by comparing the binding affinity of TFs for motifs containing either the ancestral or variant allele. atSNP computes the *p*-value for the log-rank statistic based on the best motif matches for both alleles, referred to as the *p*-value impact, with significance defined as *p* ≤ 0.01. Results were expressed as log-odds scores.

### 4.11. Clustered Regularly Interspaced Short Palindromic Repeats (CRISPR)

First, it was necessary to assess the SNV c.-1577G>A genotype of each cell line so that the gRNA could be created for each one. For cells of the SK-MEL-28 cell line, it was necessary to change to the SNV variant allele (CTTGAAGATTTCTA; where the SNV site is underlined). For the A-375 cell line, it was necessary to change to the ancestral allele (CTTGAAGGTTTCTA; where the SNV site is underlined) and the variant allele, so that the functional analyses are based on the SNV ancestral and variant of interest.

The chosen gRNA had the sequence GCGCTTGAGCTGGGGCTTGA, which indicated to Cas9 the region that should be cut. The HDR sequence (cccattaggttgttattg-CTTGTTGGCGCTTGAGCTGGGGCTTTGAAGATTTCTATAATGTGTAGCAGTGTATAGAAAACAGGCAGGTCAGAAAA) was chosen to be inserted into the cut region. To validate sequences, off-target regions were evaluated to prevent cuts in non-specific regions.

Once designed, the sequences were inserted into different plasmids for cloning. For the gRNA, the vector chosen was eSpCas9-2A-GFP (PX458), which contains Cas9 and the GFP marker region. For the HDR template, the one chosen was pUC57. Approximately 2 µg of gRNA and HDR were transfected into SK-MEL-28 and A-375 cells using Lipofectamine 3000 reagent (Invitrogen, USA) following the manufacturer’s instructions.

### 4.12. Cell Cycle and Proliferation Analysis

For cell cycle analysis, cells with different genotypes were seeded in 25cm² culture flasks (5 × 10^5^ cells per flask). After transfection, cell pellets were fixed with ice-cold 70% ethanol, incubated for 1 h at 4 °C, stained by 7-amino actinomycin D (7-AAD, Life Technologies, USA), and evaluated by the cytometer BD FACSVerse™ (BD Biosciences, USA). Cells were classified according to size (Forward Scatter, FSC, Bonn, Germany) and granularity (Side Scatter, SSC, New Delhi, India). The intensity of 7-AAD labeling and separation of cell cycle phases G1, S, and G2 were analyzed by the FCS Express Flow software Cytometry Analysis (https://denovosoftware.com/) (Thermo Fisher Scientific, Waltham, MA, USA).

For proliferation analysis, cells with different genotypes were seeded in 96-well plates (4 × 10^3^ cells per well). After 48 h, cell proliferation was quantified by Cell Counting Kit 8 (CCK-8, Sigma-Aldrich, St. Louis, MO, USA). The absorbance reading was performed at 450 nanometers (nm) using the microplate reader spectrophotometer Epoch (BioTek, Winooski, VT, USA).

### 4.13. Cell Apoptosis and Necrosis Analysis

Cells with different genotypes were cultivated in 25cm^2^ culture flasks (5 × 10^5^ cells per flask). After 24 h, apoptosis was stimulated using the immunotherapy drug ipilimumab (8 µg/mL) [14]. After 48 h of treatment, cells were stained with an Annexin V Apoptosis kit (Invitrogen, USA) and evaluated in cytometer BD FACSVerse™. The fraction of live cells, early and late apoptotic cells, and necrotic cells was determined using FlowJo™ v10.8 Software (BD Life Sciences, Franklin Lakes, NJ, USA).

### 4.14. Cell Migration Analysis

The analysis of the migratory capacity of cells with different genotypes was evaluated with the wound healing assay. For this purpose, cells were seeded in 12-well culture plates (1 × 10^5^ cells per well). After 24 h, a perpendicular straight line was drawn to remove the adhered cells, forming the “wounds”, using sterilized pipette tips. The cells were cultured in DMEM medium plus 2% FBS and 1% P/S. The wounds were photographed at the initial time (T0h) and until the wound was closed. The images were analyzed using Image J software (version number 1.54d) (National Institutes of Health, Rockville Pike, MD, USA).

### 4.15. Statistical Analyses

HWE test was performed to verify whether there was a preferential distribution of any of the *CTLA-4* genotypes in the group of patients and controls. Haploview 4.2 software (https://www.broadinstitute.org/haploview/haploview) (accessed on 16 March 2022) was used to select markers included in the haplotype analysis. Linkage disequilibrium was measured by the disequilibrium coefficient (D′), and significance was considered at D′ ≥ 0.80.

Datasets were probed for normality using the Shapiro–Wilk test. Samples did assume a normal distribution; thus, we used the t-test to compare the groups. The Bonferroni method was used to adjust the values in multiple comparisons of SNVs with the clinicopathologic aspects of patients. EFS and MSS were calculated using Kaplan–Meier curves, and differences between curves were calculated using the log-rank test. Cox univariate and multivariate analyses were used to evaluate the effect of clinicopathological and genotypic characteristics on patient survival. Variables with *p* < 0.05 were considered significant.

All analyses were performed using the statistical program RStudio (PBC, Roscoe Township, IL, USA).

## 5. Conclusions

In conclusion, our data provide, for the first time, evidence that the germline SNV c.-1577G > A alters CM risk and influences clinical aspects of CM patients. This variant may also serve as an independent prognostic factor in CM patients treated with surgical tumor resection and conventional chemotherapy. These associations may be attributed to its impact on the gene promoter region, influencing cell cycle regulation and the mechanisms of cell proliferation, survival, and migration. We believe that our study offers initial insights that warrant exploration in larger studies, with the goal of using these data to identify healthy individuals for additional preventive and early-detection measures and to guide treatment selection for CM patients.

## Figures and Tables

**Table 1:** Frequencies of the distributions of the 432 patients with cutaneous melanoma and the 504 controls according to clinical characteristics and tumor.

Variables	Patients *n* (%)	Controls *n* (%)	*p-*Value
Age (years)			
≤54	211 (48.8)	424 (84.1)	0.0001
>54	221 (51.2)	80 (15.9)	
Sex			
Male	199 (46.1)	231 (45.8)	0.99
Female	233 (53.9)	273 (54.2)	
Sun exposure *			
Yes	346 (82.4)	310 (62.1)	0.0001
No	74 (17.6)	189 (37.9)	
Skin color			
White	403 (93.3)	442 (87.7)	0.005
Brown or black	29 (6.7)	62 (12.3)	
Phototype *			
I–III	398 (95.7)	451 (90.4)	0.003
IV–VI	18 (4.3)	48 (9.6)	
Eye color *			
Blue or green	129 (31.1)	94 (18.8)	0.0001
Brown or black	286 (68.9)	405 (81.2)	
Tumor location *			
Trunk	194 (44.8)	NA	
Head or neck	77 (17.8)		
Upper limb	64 (14.8)		
Lower limb	78 (18.0)		
Histological type *			
Superficial spreading	253 (58.7)	NA	
Nodular	103 (23.8)		
Lentigo maligna	34 (7.9)		
Unspecified (non-acral)	42 (9.7)		
Clark levels *		NA	
I	38 (8.8)		
II	71 (16.4)		
III	130 (30.0)		
IV	161 (37.2)		
V	19 (4.4)		
Clinical stage *		NA	
0	36 (8.3)		
I	193 (44.6)		
II	131 (30.3)		
III	53 (12.2)		
IV	14 (3.2)		

*n*: absolute number of individuals; %: percentage; NA: not applicable * The number of individuals differs from the initial number because it was not possible to obtain the information in some cases. Skin phototype was defined as described by Fitzpatrick.

**Table 2:** Frequencies of alleles and isolated and combined *CTLA-4* genotypes in 432 patients with cutaneous melanoma and 504 controls.

Genotypes	Patients *n* (%)	Controls *n* (%)	*p-*Value	OR * (95% CI)
**c.-1765C>T**				
CC or CT	363 (84.0)	431 (85.5)	0.91	Reference
TT	69 (16.0)	73 (14.5)		1.02 (0.64–1.47)
C	534 (61.8)	632 (62.7)	0.92	Reference
T	330 (38.2)	376 (37.3)		1.01 (0.81–1.24)
**c.-1661A>G**				
AA or AG	405 (93.8)	458 (90.9)	0.24	1.39 (0.40–1.24)
GG	27 (6.2)	46 (9.1)		Reference
A	662 (76.6)	735 (72.9)	0.36	Reference
G	202 (23.4)	273 (27.1)		1.11 (0.70–1.13)
**c.-1577G>A**				
GG or GA	312 (72.2)	403 (80.0)	0.007	Reference
AA	120 (27.8)	101 (20.0)		1.60 (1.13–2.26)
G	450 (52.1)	562 (55.8)	0.10	Reference
A	414 (47.9)	446 (44.2)		1.18 (0.96–1.46)
**c.-1478G>A**				
GG or GA	394 (91.2)	458 (90.9)	0.96	NE
AA	38 (8.8)	46 (9.1)		
G	644 (74.5)	728 (72.2)	0.44	Reference
A	220 (25.5)	280 (27.8)		1.09 (0.72–1.52)
**c.-1765C>T + c.-1577G>A**				
CC or CT + GG or GA	261 (93.5)	347 (95.3)	0.92	0.95 (0.44–2.10)
TT + AA	18 (6.5)	17 (4.7)		Reference
**c.-1765C>T + c.-1577G>A**				
CC or CT + GG or GA	261 (93.5)	347 (95.3)	0.92	0.95 (0.44–2.10)
TT + AA	18 (6.5)	17 (4.7)		Reference
**c.-1577G>A + c.-1478G>A**				
GG or GA + GG or GA	385 (96.3)	364 (98.1)	0.04	Reference
AA + AA	11 (3.7)	7 (1.9)		3.12 (1.03–9.74)
**c.-1577G>A + c.-1661A>G**				
GG or GA + AA or AG	291 (98.0)	371 (96.4)	0.36	Reference
AA + GG	6 (2.0)	14 (3.6)		1.65 (0.18–1.72)
**c.-1765C>T + c.-1661A>G**				
CC or CT + AA or AG	353 (95.4)	421 (92.1)	0.91	1.02 (0.67–1.42)
TT + GG	17 (4.6)	36 (7.9)		Reference

*n*: number of cases; %: percentage; CI: confidence interval; * OR: odds ratio adjusted for age, sun exposure, skin color, and eye color; NE: not evaluated.

**Table 3:** Frequencies of isolated genotypes of the *CTLA-4* variants in 432 patients with cutaneous melanoma, stratified by clinical aspects.

Genotypes	Age		Sex		Sun Exposure *		Skin Color *		Eye Color *	
	≤54 *n* (%)	>54 *n* (%)	Male *n* (%)	Female *n* (%)	Yes *n* (%)	No *n* (%)	White *n* (%)	Others *n* (%)	Blue/Green *n* (%)	Others *n* (%)
**c.-1765C>T**										
CC	79 (37.4)	92 (41.6)	83 (41.7)	88 (51.5)	49 (49.5)	118 (37.3)	138 (39.8)	30 (40.5)	64 (49.6)	103 (50.8)
CT or TT	132 (62.6)	129 (58.4)	116 (59.3)	145 (55.6)	50 (50.5)	198 (62.6)	209 (60.2)	44 (59.5)	65 (50.4)	183 (49.2)
*p-*value	0.42		0.46		1.00		0.24		1.00	
CC or CT	188 (89.1)	175 (79.2)	166 (83.4)	197 (84.5)	87 (87.9)	262 (82.9)	287 (82.7)	64 (86.5)	112 (86.8)	237 (82.9)
TT	23 (10.9)	46 (20.8)	34 (16.6)	36 (15.5)	12 (12.1)	54 (17.1)	59 (17.3)	10 (13.5)	17 (13.2)	49 (17.1)
*p-*value	0.007 *		0.85		0.56		0.64		0.56	
**c.-1661A>G**										
AA	119 (56.4)	138 (62.4)	133 (66.8)	124 (53.2)	57 (75.4)	187 (76.6)	209 (60.2)	40 (54.0)	76 (68.9)	168 (69.0)
AG or GG	92 (43.6)	83 (37.6)	66 (33.2)	109 (47.7)	42 (24.6)	129 (23.4)	137 (39.8)	34 (46.0)	53 (31.1)	118 (31.0)
*p-*value	0.23		0.005 *		0.37		0.92		1.00	
AA or AG	199 (94.3)	206 (93.2)	188 (94.5)	217 (93.1)	93 (76.9)	296 (76.1)	325 (93.6)	68 (91.9)	124 (96.1)	265 (92.6)
GG	12 (5.7)	15 (6.8)	11 (5.5)	16 (6.9)	6 (23.1)	20 (23.9)	21 (6.4)	6 (8.1)	5 (3.9)	21 (7.4)
*p-*value	0.78		0.70		0.69		0.80		0.25	
**c.-1577G>A**										
GG	68 (32.2)	70 (31.7)	62 (31.5)	76 (32.6)	25 (19.1)	106 (26.1)	115 (33.1)	20 (27.0)	31 (23.7)	100 (34.5)
GA or AA	143 (67.8)	151 (68.3)	137 (68.5)	157 (67.4)	74 (80.9)	210 (73.9)	231 (66.9)	54 (73.0)	98 (76.3)	186 (65.5)
*p-*value	0.98		0.82		0.36		0.61		0.03	
GG or GA	154 (73.0)	158 (71.5)	144 (72.4)	168 (72.1)	68 (73.3)	231 (77.3)	254 (73.2)	51 (68.9)	88 (29.4)	211 (70.6)
AA	57 (27.0)	63 (28.5)	55 (27.6)	65 (27.9)	31 (26.7)	85 (22.7)	92 (26.8)	23 (31.1)	41 (35.3)	75 (64.7)
*p-*value	0.81		1.00		0.52		0.84		0.29	
**c.-1478G>A**										
GG	122 (57.8)	128 (57.9)	121 (60.8)	129 (55.4)	60 (78.1)	177 (74.7)	232 (66.8)	18 (24.3)	68 (28.7)	169 (71.3)
GA or AA	89 (42.2)	93 (42.1)	78 (39.2)	104 (44.6)	39 (21.9)	139 (25.3)	171 (33.2)	11 (75.7)	61 (33.3)	117 (65.7)
*p-*value	1.00		0.29		0.49		0.77		0.26	
GG or GA	196 (92.9)	198 (89.6)	183 (91.9)	211 (90.5)	89 (73.0)	289 (76.5)	310 (89.3)	28 (37.8)	117 (31.0)	261 (69.0)
AA	15 (7.1)	23 (10.4)	16 (8.1)	22 (9.5)	10 (27.0)	27 (23.5)	37 (10.7)	1 (2.6)	12 (32.4)	25 (67.6)
*p-*value	0.29		0.73		0.78		0.47		1.00	

*n*: number of cases; %: percentage. * The number differed from the total number (*n* = 432) because it was not possible to obtain specific information from some patients.

**Table 4:** Event-free survival and melanoma-specific survival of the 411 patients with cutaneous melanoma.

Characteristics	Univariate Analysis						Multivariate Analysis			
	N Events/N Total	EFSHR (95% CI)	*p-*Value	N Events/N Total	MSS HR (95% CI)	*p-*Value	EFS HR (95% CI)	*p-*Value	MSS HR (95% CI)	*p-*Value
**Age (years)**										
≤54	41/203	Reference	0.003	27/203	Reference	0.0003	Reference	0.01	Reference	0.001
>54	67/208	1.80 (1.21–2.65)		53/208	2.33 (1.46–3.71)		1.75 (1.11–2.75)		2.44 (1.42–4.18)	
**Sex**										
Male	48/191	1.09 (0.75–1.60)	0.62	31/191	1.43 (0.91–2.24)	0.11		NE		NE
Female	60/220	Reference		49/220	Reference					
Clinical stage *										
0 or I or II	70/338	Reference	<0.0001	51/338	Reference	<0.0001	Reference	0.0004	Reference	<0.0001
III or IV	35/66	3.43 (2.88–5.16)		29/66	3.78 (2.39–5.96)		2.39 (1.47–3.90)		3.06 (1.78–5.25)	
Histological type *										
Superficial spreading	30/247	Reference	<0.0001	20/247	Reference	<0.0001	Reference	<0.0001	Reference	<0.0001
Others	62/133	4.87 (3.14–7.54)		49/133	5.56 (3.30–9.36)		3.46 (2.20–5.45)		3.84 (2.26–6.51)	
Clark levels *										
I or II	9/106	Reference	<0.0001	5/106	Reference	0.0001	Reference	0.003	Reference	0.006
III to V	91/291	4.37 (2.20–8.68)		70/291	5.94 (2.39–14.74)		3.00 (1.42–6.32)		4.19 (1.49–11.75)	
**c.-1765C>T**										
CC	47/162	Reference	0.36	36/162	Reference	0.28		NE		NE
CT or TT	61/249	0.83 (0.57–1.22)		44/249	0.78 (0.50–1.22)					
CC or CT	93/346	Reference	0.59	70/346	Reference	0.41		NE		NE
TT	15/65	0.86 (0.49–1.48)		10/65	0.76 (0.39–1.47)					
**c.-1661A>G**										
AA	68/246	Reference	0.57	51/246	Reference	0.58		NE		NE
AG or GG	40/165	0.89 (0.60–1.32)		29/165	0.87 (0.55–1.38)					
AA or AG	104/387	Reference	0.30	77/387	Reference	0.42		NE		NE
GG	4/24	0.59 (0.21–1.61)		3/24	0.62 (0.19–1.98)					
**c.-1577G>A**										
GG	32/127	Reference	0.84	25/127	Reference	0.85		NE	Reference	0.20
GA or AA	76/284	1.04 (0.68–1.57)		55/284	0.95 (0.59–1.53)				1.61 (0.29–1.29)	
GG or GA	69/294	Reference	0.02	51/294	Reference	0.04	Reference	0.01	Reference	0.06
AA	39/117	1.60 (1.06–2.32)		29/117	1.60 (1.00–2.50)		1.75 (1.10–2.78)		1.67 (0.98–2.84)	
**c.-1478G>A**										
GG	70/239	Reference	0.12	52/239	Reference	0.22	Reference	0.10		NE
GA or AA	38/172	1.36 (0.49–1.08)		28/172	0.75 (0.47–1.19)		1.44 (0.44–1.07)			
GG or GA	100/374	0.80 (0.39–1.65)	0.55	75/374	0.69 (0.28–1.72)	0.43		NE		NE
AA	8/37	Reference		5/37	Reference					

N: number of patients; EFS: event-free survival; MSS: melanoma-specific survival; HR: hazzard ratio of events; CI: confidence interval; NE: not evaluated. * The number differed from the total number (*n* = 411) because it was not possible to obtain specific information from some patients. Factors with *p-*values ≤ 0.10 were included in the Cox multivariate analysis. Factors with *p-*values < 0.05 were considered significant.

## Data Availability

The data that support the findings of this study are available from the corresponding author upon reasonable request.

## Supplementary Materials

The following supporting information can be downloaded at: https://www.mdpi.com/article/10.3390/ijms252212327/s1.
