# Supplementary material for: Association of Cytotoxic T-Lymphocyte Antigen-4 (CTLA-4) Genetic Variants with Risk and Outcome of Cutaneous Melanoma
Source: Int J Mol Sci. 2024 Nov 17;25(22):12327. doi: 10.3390/ijms252212327 (PMC11594856; doi:10.3390/ijms252212327)
Supplement: Supplementary file 1 [file ijms-25-12327-s001.zip › ijms-3290036-supplementary.pdf]

Article - Supplementary Material

Association of Cytotoxic T-Lymphocyte Antigen-4 (*CTLA-4*) Genetic Variants with Risk and Outcome of Cutaneous Melanoma

Ana Maria Castro Ferreira [1], Juliana Carron [1,2], Gabriela Vilas Bôas Gomez [1], Vinicius de Lima Vazquez [3], Sergio Vicente Serrano [4], Gustavo Jacob Lourenço [1] and Carmen Silvia Passos Lima [1,2,*]

1 Laboratory of Cancer Genetics, School of Medical Sciences, State University of Campinas, Campinas 13083-970, SP, Brazil; castroferreiraana@gmail.com (A.M.C.F.); jcarron@unicamp.br (J.C.); gabivbg@gmail.com (G.V.B.G.); guslour@unicamp.br (G.J.L.)
2 Department of Anesthesiology, Oncology and Radiology, School of Medical Sciences, State University of Campinas, Campinas 13083-970, SP, Brazil
3 Melanoma and Sarcoma Surgery Department, Barretos Cancer Hospital, Barretos 14784-400, SP, Brazil; viniciusvazquez@gmail.com
4 Department of Medical Oncology, Barretos Cancer Hospital, Barretos 14784-400, SP, Brazil; svserrano@hotmail.com
* Correspondence: carmenl@fcm.unicamp.br; Tel.: +55-19-3521-9120

Table S1. Haplotype frequencies of *CTLA-4* variants in 432 patients with cutaneous melanoma and 504 controls.

c.-1765C>T	c.-1661A>G	c.-1577G>A	c.1478G>A	Patients	Controls	*p*-value	OR* (95% CI)
C	A	A	G	183	209	0.98	1.00 (0.74-1.34)
T	G	A	G	193	192	0.89	1.02 (0.72-1.45)
C	G	A	G	193	192	0.08	0.77 (0.57-1.03)

*OR: odds ratio adjusted for age, sun exposure, skin color, and eye color; CI: confidence interval.

Table S2. Frequencies of combined genotypes of the *CTLA-4* variants in 432 patients with cutaneous melanoma stratified by clinical aspects.

Genotypes	Median age		Sex		Sun exposure*		Skin color*		Eye color*	
	≤ 54 n (%)	> 54 n (%)	Male n (%)	Female n (%)	Yes n (%)	No n (%)	White n (%)	Others n (%)	Blue/green n (%)	Others n (%)
**c.-1765C>T + c.-1577G>A**										
CC or CT + GG or GA	136 (52.1)	5 (27.8)	120 (93.0)	141 (94.0)	213 (75.3)	41 (100.0)	246 (94.3)	15 (94.4)	78 (31.1)	7 (38.9)
TT + AA	125 (47.9)	13 (72.2)	9 (7.0)	9 (6.0)	18 (24.7)	0 (0.0)	17 (5.7)	1 (5.6)	173 (68.9)	11 (61.1)
p-value	0.07		0.93		0.13		1.00		0.66	
CC + GG	16 (49.4)	19 (54.3)	16 (46.2)	19 (54.3)	34 (14.7)	1 (24.3)	33 (7.6)	2 (5.7)	10 (29.4)	24 (29.1)
CT or TT + GA or AA	80 (50.6)	78 (45.7)	85 (53.8)	73 (45.7)	127 (85.3)	25 (75.7)	146 (92.4)	12 (94.3)	44 (70.6)	107 (70.9)
p-value	0.73		0.49		0.06		0.97		1.00	
**c.-1765C>T + c.-1478G>A**										
CC or CT + GG or GA	174 (52.3)	159 (47.7)	152 (45.6)	181 (54.4)	266 (72.4)	55 (66.7)	310 (93.1)	23 (6.9)	102 (31.9)	218 (68.1)
TT + AA	1 (12.5)	7 (87.5)	2 (25.0)	6 (75.0)	6 (27.6)	2 (33.3)	7 (87.5)	1 (12.5)	2 (25.0)	6 (75.0)
p-value	0.06		0.42		0.72		1.00		0.97	
CC + GG	45 (50.6)	44 (49.4)	45 (50.6)	44 (49.4)	69 (29.9)	17 (41.5)	84 (94.4)	5 (5.6)	30 (34.9)	56 (65.1)
CT or TT + GA or AA	55 (55.0)	45 (45.0)	61 (61.0)	39 (39.0)	76 (70.1)	20 (58.5)	92 (92.0)	8 (8.0)	27 (27.8)	70 (72.2)
p-value	0.64		0.19		1.00		0.72		0.38	
**c.-1765C>T + c.-1661A>G**										
CC or CT + AA or AG	182 (69.7)	171 (64.7)	191 (54.1)	162 (45.9)	279 (24.1)	62 (75.9)	330 (93.5)	23 (6.5)	110 (32.4)	230 (67.6)
TT + GG	6 (30.3)	11 (35.3)	10 (58.8)	7 (41.2)	13 (5.9)	4 (94.1)	16 (94.1)	1 (5.9)	3 (17.6)	14 (82.4)
p-value	0.28		0.89		0.14		1.00		0.31	
CC + AA	59 (48.4)	63 (57.1)	60 (35.7)	62 (50.8)	98 (26.9)	21 (73.1)	115 (43.7)	7 (43.7)	43 (36.1)	76 (63.9)
CT or TT + AG or GG	72 (51.6)	54 (42.9)	81 (64.3)	45 (49.2)	97 (20.3)	25 (79.7)	116 (56.3)	10 (56.3)	32 (26.0)	91 (74.0)
p-value	0.20		0.02		0.69		0.66		0.11	
**c.-1577G>A + c.-1478G>A**										
GG or GA + GG or GA	144 (50.5)	141 (49.5)	131 (46.0)	154 (54.0)	62 (22.7)	211 (77.3)	266 (93.3)	19 (6.7)	81 (29.7)	192 (70.3)
AA + AA	5 (45.5)	6 (54.5)	3 (27.3)	8 (72.7)	4 (36.4)	7 (63.6)	11 (100.0)	0 (0.0)	5 (45.5)	6 (54.5)
p-value	0.98		0.36		0.49		0.79		0.43	
GG + GG	24 (40.0)	36 (60.0)	36 (60.0)	24 (40.0)	234 (21.1)	44 (78.9)	55 (91.7)	5 (8.3)	13 (22.8)	44 (77.2)
GA or AA + GA or AA	45 (43.3)	59 (56.7)	64 (61.5)	40 (38.5)	7 (25.0)	4 (75.0)	99 (95.2)	5 (4.8)	43 (41.3)	61 (58.7)
p-value	0.80		0.97		0.16		0.56		0.02	

**c.-1577G>A + c.-1661A>G**										
GG or GA + AA or AG	145 (49.8)	146 (50.2)	135 (46.4)	156 (53.6)	51 (22.9)	9 (77.1)	272 (93.5)	19 (6.5)	84 (30.1)	195 (69.9)
AA + GG	3 (50.0)	3 (50.0)	2 (33.3)	4 (66.7)	81 (33.3)	22 (66.7)	6 (100.0)	0 (0.0)	1 (16.7)	5 (83.3)
p-value	1.00		0.82		0.16		1.00		0.79	
GG + AA	25 (39.1)	39 (60.9)	33 (51.6)	31 (48.4)	51 (18.0)	9 (82.0)	58 (90.6)	6 (9.4)	17 (27.9)	44 (72.1)
GA or AA + AG or GG	49 (48.5)	52 (51.5)	66 (65.3)	35 (34.7)	81 (27.7)	22 (72.3)	95 (94.1)	6 (5.9)	39 (38.6)	62 (61.4)
p-value	0.30		0.11		0.42		0.60		0.22	
**c.-1478G>A + c.-1661A>G**										
GG or GA + AA or AG	191 (50.0)	191 (50.0)	205 (53.7)	177 (46.3)	88 (24.0)	278 (76.0)	354 (92.7)	28 (7.3)	115 (31.4)	251 (68.6)
AA + GG	7 (46.7)	8 (53.3)	10 (66.7)	5 (33.3)	5 (35.7)	9 (64.3)	14 (93.3)	1 (6.7)	3 (21.4)	11 (78.6)
p-value	1.00		0.46		0.49		1.00			0.61
GG + AA	100 (47.8)	109 (52.2)	101 (48.3)	108 (51.7)	49 (25.0)	147 (75.0)	194 (92.8)	15 (7.2)	57 (29.1)	139 (70.9)
GA or AA + AG or GG	70 (52.2)	64 (47.8)	81 (60.4)	53 (39.6)	31 (23.8)	99 (76.2)	126 (94.0)	8 (6.0)	42 (32.3)	88 (67.7)
p-value	0.49		0.03		0.91		0.82		0.61	

n: number of cases; %: percentage; CI: confidence interval; *OR: odds ratio adjusted for age. skin color. eye color and sun exposure. *the number differed from the total number (n= 432) because it was not possible to obtain specific information from some patients. *Significant *P*-value after Bonferroni correction (*P*< 0.01).

Table S3. Frequencies of isolated and combined genotypes of variants in the *CTLA-4* gene in patients with cutaneous melanoma stratified by tumor aspects.

Genotypes	Tumor location*		Clark levels*		Tumor stage*		Histological type*	
	Head, limbs n (%)	Trunk n (%)	I-II n (%)	III-V n (%)	I-II n (%)	III-IV n (%)	Superficial n (%)	Others n (%)
**c.-1765C>T**								
CC	67 (66.3)	34 (33.7)	38 (23.8)	122 (76.2)	122 (79.7)	31 (20.3)	95 (61.7)	59 (38.3)
CT or TT	107 (67.9)	49 (31.4)	67 (27.0)	181 (73.0)	183 (79.9)	46 (20.1)	156 (66.1)	80 (33.9)
p-value	0.80		0.53		1.00		0.43	
CC or CT	145 (67.4)	70 (32.6)	90 (26.0)	256 (74.0)	256 (80.0)	64 (20.0)	212 (64.8)	115 (35.2)
TT	29 (69.0)	13 (31.0)	15 (24.2)	47 (75.8)	49 (79.0)	13 (21.0)	39 (61.9)	24 (38.1)
p-value	0.98		0.88		0.99		0.76	
**c.-1577G>A**								
GG	37 (59.7)	25 (40.3)	30 (23.1)	100 (76.9)	99 (79.2)	26 (20.8)	84 (66.7)	42 (33.3)
GA or AA	137 (70.3)	58 (29.7)	75 (27.0)	203 (73.0)	206 (80.2)	51 (19.8)	167 (63.3)	97 (36.7)
p-value	0.16		0.47		0.93		0.58	
GG or GA	109 (65.7)	57 (34.3)	75 (25.4)	220 (74.6)	224 (80.0)	56 (20.0)	187 (65.6)	98 (34.4)
AA	65 (71.4)	26 (28.6)	30 (26.5)	83 (73.5)	81 (79.4)	21 (20.6)	64 (61.0)	41 (39.0)
p-value	0.42		0.91		1.00		0.46	
**c.-1661A>G**								
AA	123 (71.9)	48 (28.1)	61 (25.1)	182 (74.9)	180 (78.6)	49 (21.4)	145 (63.0)	85 (37.0)
AG or GG	51 (59.3)	35 (40.7)	44 (26.7)	121 (73.3)	125 (81.7)	28 (18.3)	106 (66.2)	54 (33.8)
p-value	0.05		0.81		0.54		0.58	
AA or AG	168 (68.6)	77 (31.4)	98 (25.6)	285 (74.4)	286 (80.1)	71 (19.9)	233 (64.2)	130 (35.8)
GG	6 (50.0)	6 (50.0)	7 (28.0)	18 (72.0)	19 (76.0)	6 (24.0)	18 (66.7)	9 (33.3)
p-value	0.30		0.97		0.81		0.95	
**c.-1478G>A**								
GG	126 (72.4)	48 (27.6)	63 (26.9)	171 (73.1)	177 (80.1)	44 (19.9)	143 (64.1)	80 (35.9)
GA or AA	48 (57.8)	35 (42.2)	42 (24.1)	132 (75.9)	128 (79.5)	33 (20.5)	108 (64.7)	59 (35.3)
p-value	0.02		0.60		0.99		0.99	
GG or GA	167 (68.4)	77 (31.6)	96 (25.8)	276 (74.2)	278 (80.1)	69 (19.9)	228 (64.8)	124 (35.2)

AA	7 (53.8)	6 46.2)	9 (25.0)	27 (75.0)	27 (19.9)	8 (22.9)	23 (60.5)	15 (39.5)
p-value	0.42		1.00		0.84		0.73	
**c.-1765C>T + c.-1577G>A**								
CC or CT + GG or GA	92 (67.2)	45 (32.8)	62 (24.8)	188 (755.2)	186 (79.8)	47 (20.2)	160 (67.2)	78 (32.8)
TT + AA	12 (92.3)	1 (7.7)	2 (11.8)	15 (88.2)	11 (73.3)	4 (26.7)	12 (75.0)	4 (25.0)
p-value	0.11		0.35		0.78		0.71	
CC + GG	2 (40.0)	3 (60.0)	4 (11.8)	30 (88.2)	24 (70.6)	10 (29.4)	24 (68.6)	11 (31.4)
CT or TT + GA or AA	72 (72.7)	27 (27.3)	41 (27.0)	111 (73.0)	108 (78.3)	30 (21.7)	96 (66.2)	49 (33.8)
p-value	0.28		0.09		0.47		0.94	
**c.-1765C>T + c.-1478G>A**								
CC or CT + GG or GA	141 (68.8)	64 (31.2)	81 (25.6)	236 (74.4)	233 (80.1)	58 (19.9)	194 (65.3)	103 (34.7)
TT + AA	3 (100.0)	0 (0.0)	0 (0.0)	7 (100.0)	4 (66.7)	2 (33.3)	5 (62.5)	3 (37.5)
p-value	0.59		0.27		0.76		1.00	
CC + GG	41 (74.5)	14 (25.5)	19 (22.9)	64 (77.1)	67 (82.7)	14 (17.3)	50 (63.3)	29 (36.7)
CT or TT + GA or AA	22 (59.5)	15 (40.5)	23 (23.7)	74 (76.3)	73 (82.0)	16 (18.0)	63 (68.5)	29 (31.5)
p-value	0.19		1.00		1.00		0.58	
**c.-1765C>T + c.-1661A>G**								
CC or CT + AA or AG	141 (68.8)	64 (31.2)	87 (25.9)	249 (74.1)	250 (80.4)	61 (19.6)	205 (64.7)	112 (35.3)
TT + GG	2 (100.0)	0 (0.0)	4 (26.7)	11 (73.3)	13 (81.2)	3 (18.8)	11 (64.7)	6 (35.3)
p-value	0.85		1.00		1.00		1.00	
CC + AA	39 (73.6)	14 (26.4)	25 (21.6)	91 (78.4)	94 (81.7)	21 (18.3)	74 (66.1)	38 (33.9)
CT or TT + AG or GG	23 (60.5)	15 (39.5)	31 (25.6)	90 (74.4)	97 (84.3)	18 (15.7)	85 (72.0)	33 (28.0)
p-value	0.27		0.55		0.72		0.40	
**c.-1577G>A + c.-1478G>A**								
GG or GA + GG or GA	105 (66.0)	54 (34.0)	70 (25.9)	200 (74.1)	205 (80.4)	50 (19.6)	172 (66.7)	86 (33.3)
AA + AA	3 (50.0)	3 (50.0)	4 (36.4)	7 (63.6)	8 (80.0)	2 (20.0)	8 (72.7)	3 (27.3)
p-value	0.70		0.67		1.00		0.92	
GG + GG	18 (60.0)	12 (40.0)	13 (23.2)	43 (76.8)	47 (83.9)	9 (16.1)	39 (69.6)	17 (30.4)
GA or AA + GA or AA	29 (56.9)	22 (43.1)	25 (25.0)	75 (75.0)	76 (82.6)	16 (17.4)	63 (64.9)	34 (35.1)
p-value	0.96		0.95		1.00		0.67	
**c.-1577G>A + c.-1661A>G**								
GG or GA + AA or AG	105 (66.0)	54 (34.0)	70 (25.4)	206 (74.6)	211 (80.8)	50 (19.2)	173 (65.5)	91 (34.5)
AA + GG	2 (40.0)	3 (60.0)	2 (33.3)	4 (66.7)	6 (100.0)	0 (0.0)	4 (66.7)	2 (33.3)
p-value	0.46		1.00		0.50		1.00	
GG + AA	17 (58.6)	12 (41.4)	14 (23.7)	45 (76.3)	47 (77.0)	14 (23.0)	38 (63.3)	22 (36.7)
GA or AA + AG or GG	31 (58.5)	22 (41.5)	28 (29.8)	66 (70.2)	73 (82.0)	16 (18.0)	60 (63.8)	34 (36.2)
p-value	1.00		0.52		0.58		1.00	

**c.-1478G>A + c.-1661A>G**								
GG or GA + AA or AG	166 (68.3)	77 (31.7)	92 (25.5)	269 (74.5)	267 (79.7)	68 (20.3)	219 (64.6)	121 (35.6)
AA + GG	5 (45.5)	6 (54.5)	3 (21.4)	11 (78.6)	8 (61.5)	5 (38.5)	9 (60.0)	6 (40.0)
p-value	0.21		0.97		0.21		0.94	
GG + AA	122 (71.8)	48 (28.2)	53 (27.0)	143 (73.0)	143 (78.6)	39 (21.4)	110 (60.4)	72 (39.6)
GA or AA + AG or GG	47 (57.3)	35 (42.7)	34 (26.8)	93 (73.2)	91 (79.8)	23 (20.2)	73 (61.3)	46 (38.7)
p-value	0.03		1.00		0.91		0.97	

n: number of cases; %: percentage; CI: confidence interval; *OR: odds ratio adjusted for age. skin color. eye color and sun exposure. *The number differed from the total number (n= 411) because it was not possible to obtain specific information from some patients. *Significant *p*-value after Bonferroni correction ($p< 0.01$).

Table S4. Event-free survival and melanoma-specific survival of the 411 patients with cutaneous melanoma stratified by combined genotypes of the *CTLA-4* variants.

Characteristics	Univariate analysis						Multivariate analysis			
	**n events/ n total**	**EFS HR (95% CI)**	***p*-value**	**n events/ n total**	**MSS HR (95% CI)**	***p*-value**	**EFS HR (95% CI)**	***p*-value**	**MSS HR (95% CI)**	***p*-value**
**c.-1765C>T + c.-1577G>A**										
CC or CT + GG or GA	60/247	Reference	0.28	46/247	Reference	0.20		NE		NE
TT + AA	6/18	1.58 (0.68-3.67)		5/18	1.81 (0.71-4.58)					
CC + GG	8/30	Reference	0.73	7/30	Reference	0.29		NE		NE
CT or TT + GA or AA	37/152	0.87 (0.40-1.88)		26/152	0.63 (0.27-1.47)					
**c.-1765C>T + c.-1478G>A**										
CC or CT + GG or GA	85/317		NE	65/317		NE		NE		NE
TT + AA	0/8			0/8						
CC + GG	27/86	Reference	0.07	22/86	Reference	0.09	Reference	0.47	Reference	NE
CT or TT + GA or AA	18/96	0.57 (0.31-1.05)		14/96	0.56 (0.28-1.10)		1.30 (0.62-2.74)		1.48 (0.61-3.55)	
**c.-1765C>T + c.-1661A>G**										
CC or CT + AA or AG	89/337		NE	67/337		NE		NE		NE
TT + GG	0/15			0/15						
CC + AA	31/116	Reference	0.33	26/116	Reference	0.28		NE		NE
CT or TT + AG or GG	24/119	0.76 (0.45-1.30)		19/119	0.72 (0.40-1.30)					
**c.-1577G>A + c.-1478G>A**										
GG or GA + GG or GA	63/268	Reference	0.72	47/268	Reference	0.49		NE		NE
AA + AA	2/11	0.77 (0.18-3.16)		1/11	0.50 (0.06-3.66)					
GG + GG	15/57	0.74 (0.38-1.44)	0.38	10/57	0.71 (0.31-1.63)	0.43		NE		NE
GA or AA + GA or AA	21/102	Reference		13/102	Reference					
**c.-1577G>A + c.-1661A>G**										
GG or GA + AA or AG	66/276	Reference	0.77	49/276	Reference	0.98		NE		NE
AA + GG	1/6	0.75 (0.10-5.43)		1/6	1.02 (0.14-7.43)					
GG + AA	14/59	0.97 (0.49-1.90)	0.93	10/59	Reference	0.73		NE		NE
GA or AA + AG or GG	22/97	Reference		14/97	0.87 (0.38-1.96)					
**c.-1478G>A + c.-1661A>G**										
GG or GA + AA or AG	100/364	Reference	0.97	75/364	Reference	0.96		NE		NE
AA + GG	4/14	1.01 (0.37-2.77)		3/14	1.02 (0.32-3.25)					
GG + AA	62/201	Reference	0.34	45/201	Reference	0.37	Reference	0.07	Reference	0.09
GA or AA + AG or GG	32/127	0.81 (0.53-1.24)		22/127	0.79 (0.47-1.31)		2.36 (0.91-6.15)		2.92 (0.83-10.22)	

n: number of patients; EFS: event-free survival; MSS: melanoma-specific survival; HR: hazard ratio of events; CI: confidence interval; NE: not evaluated. Factors with *p*-values ≤ 0.10 were included in the Cox multivariate analysis. Factors with *p*-values < 0.05 were considered significant.

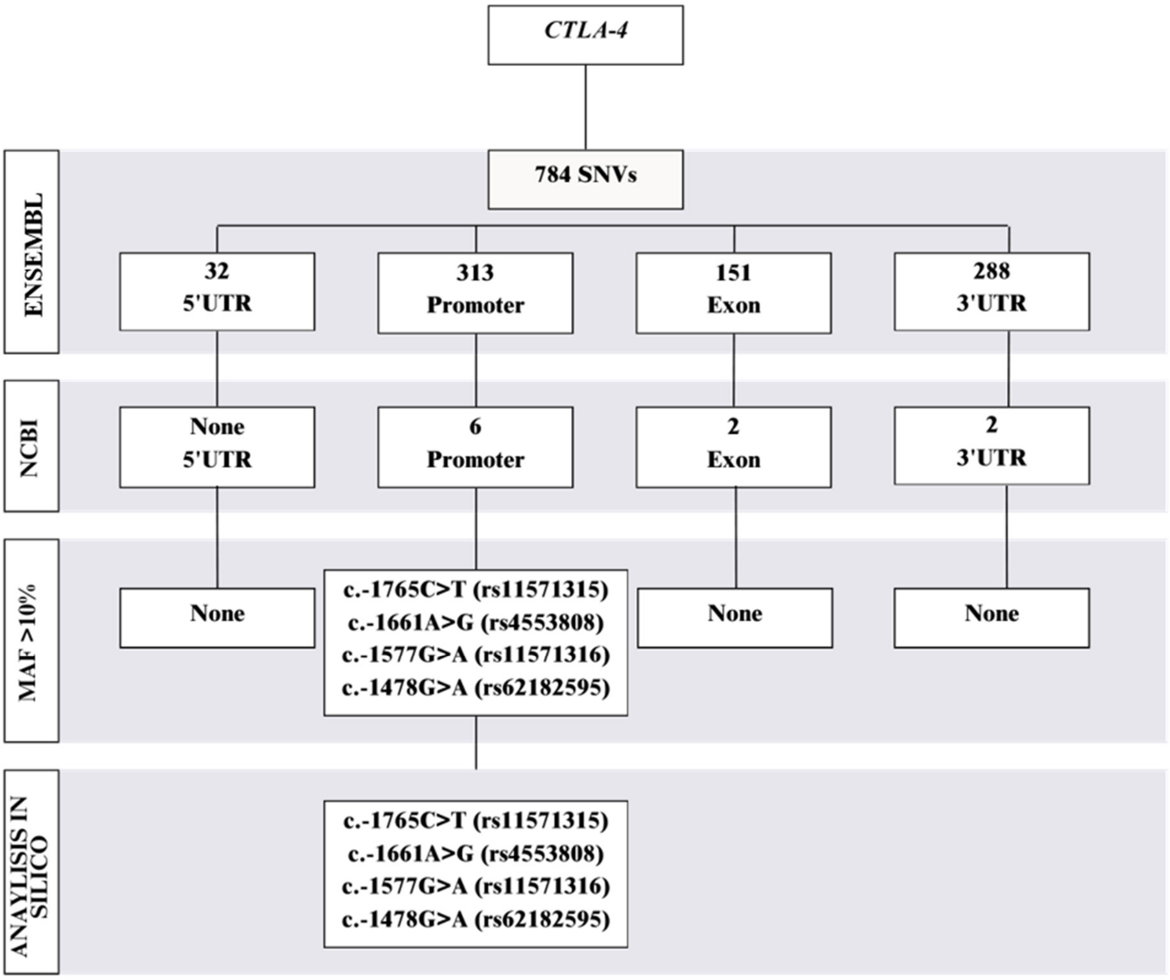

Figure S1. Flowchart of the methods for selecting single nucleotide variants (SNVs) in the *CTLA-4* gene and the respective numbers of SNVs inserted at each stage. ENSEMBL: Ensembl genome database project. NCBI: National Center for Biotechnology Information. MAF: minimum allele frequency.

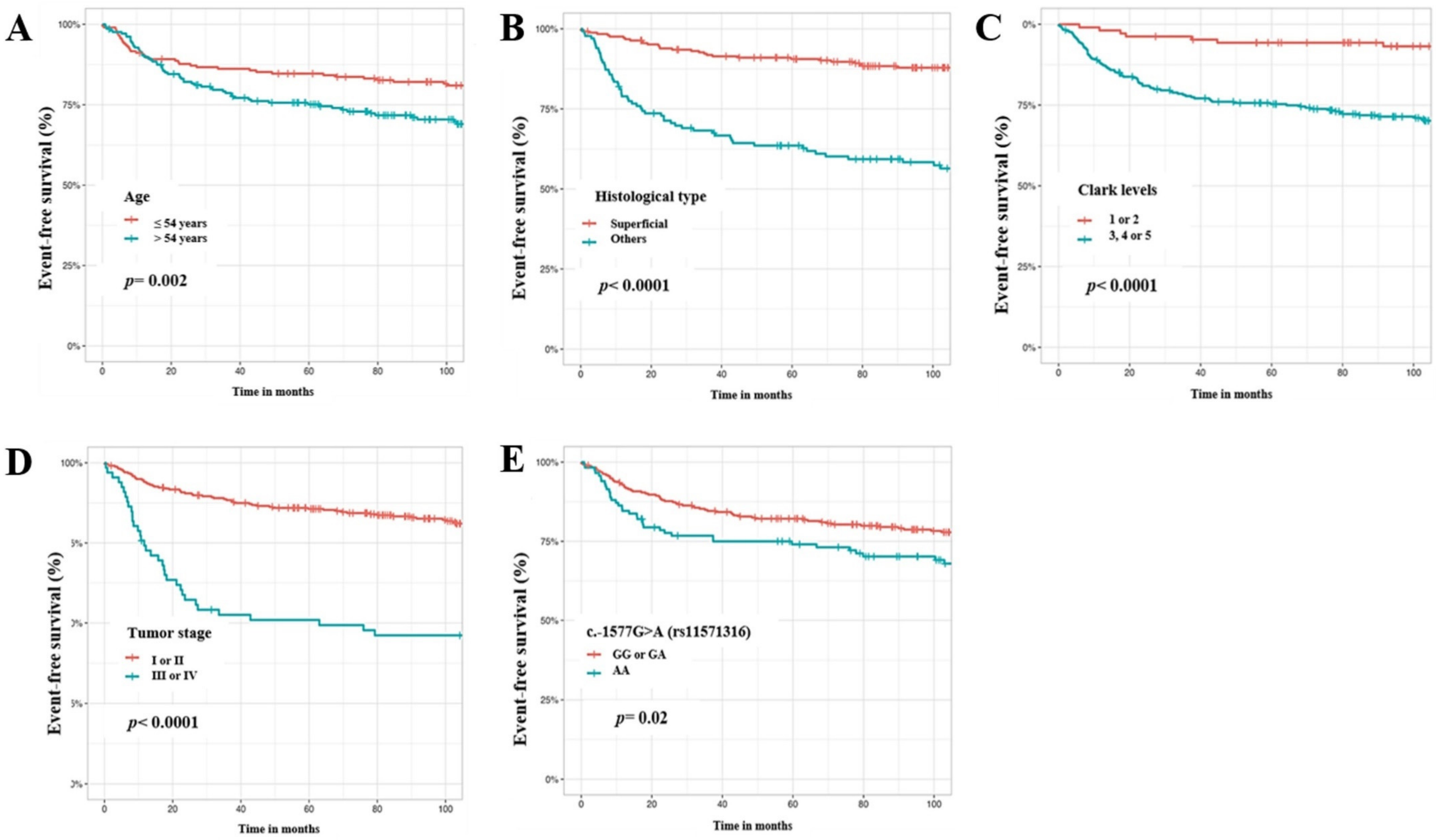
A
Age
≤ 54 years
> 54 years
p= 0.002
B
Histological type
Superficial
Others
p< 0.0001
C
Clark levels
1 or 2
3, 4 or 5
p< 0.0001
D
Tumor stage
I or II
III or IV
p< 0.0001
E
c.-1577G>A (rs11571316)
GG or GA
AA
p= 0.02
Event-free survival (%)
Time in months

Figure S2. Analysis of clinicopathologic aspects in progression-free survival in patients with cutaneous melanoma. Kaplan Meier curves indicating lower survival in patients with age greater than 54 years (A), non-superficial histological subtype (B), Clark levels III to V (C), stage III or IV (D), and *CTLA-4* c.-1577 AA genotype than in other patients (E).

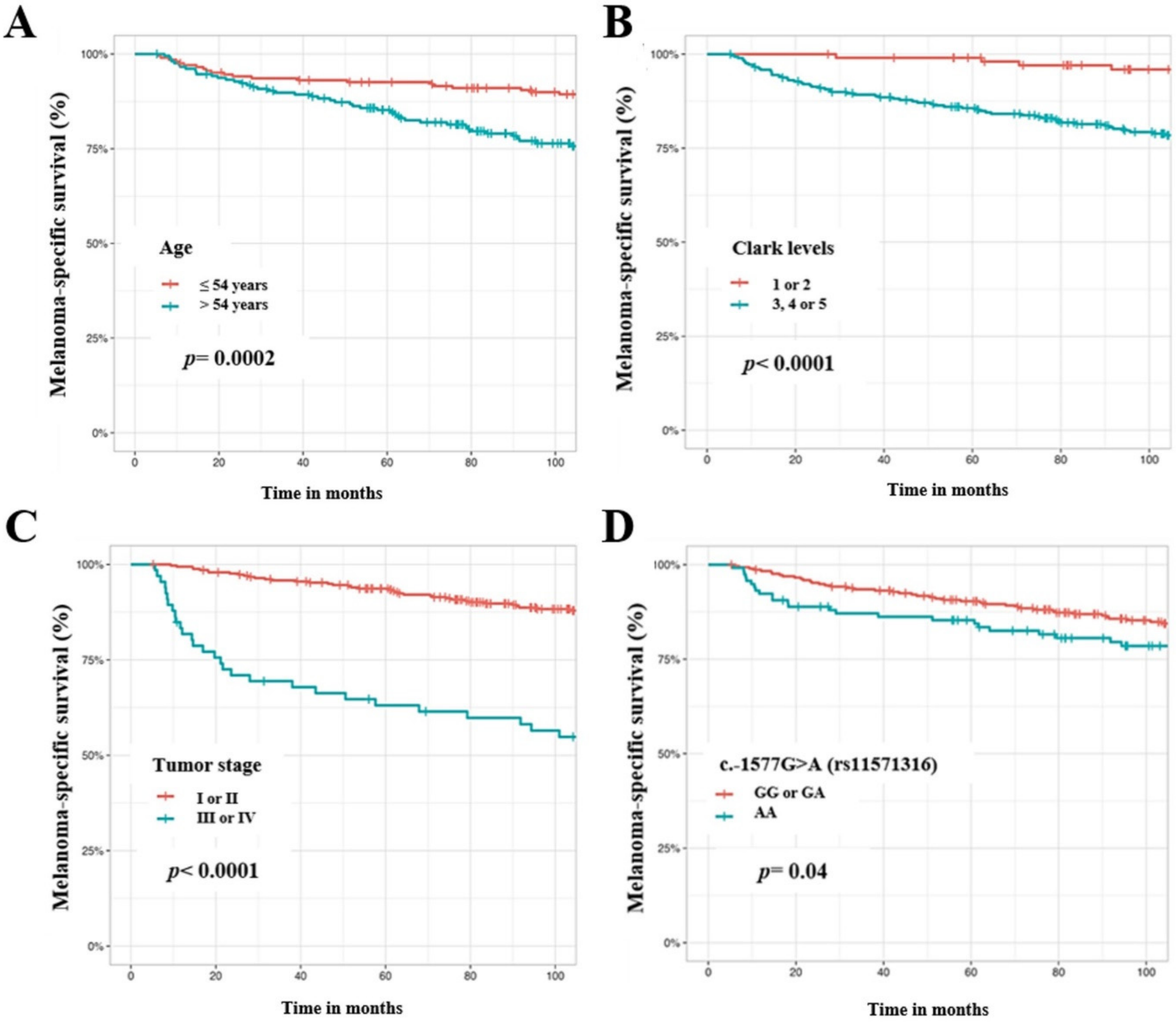

Figure S3. Analysis of clinicopathologic aspects in melanoma-specific survival in patients with cutaneous melanoma. Kaplan Meier curves indicating lower survival in patients with age greater than 54 years (A), Clark levels III or V (B), stage III or IV (C), and *CTLA-4* c.-1577 AA genotype than in other patients (D).

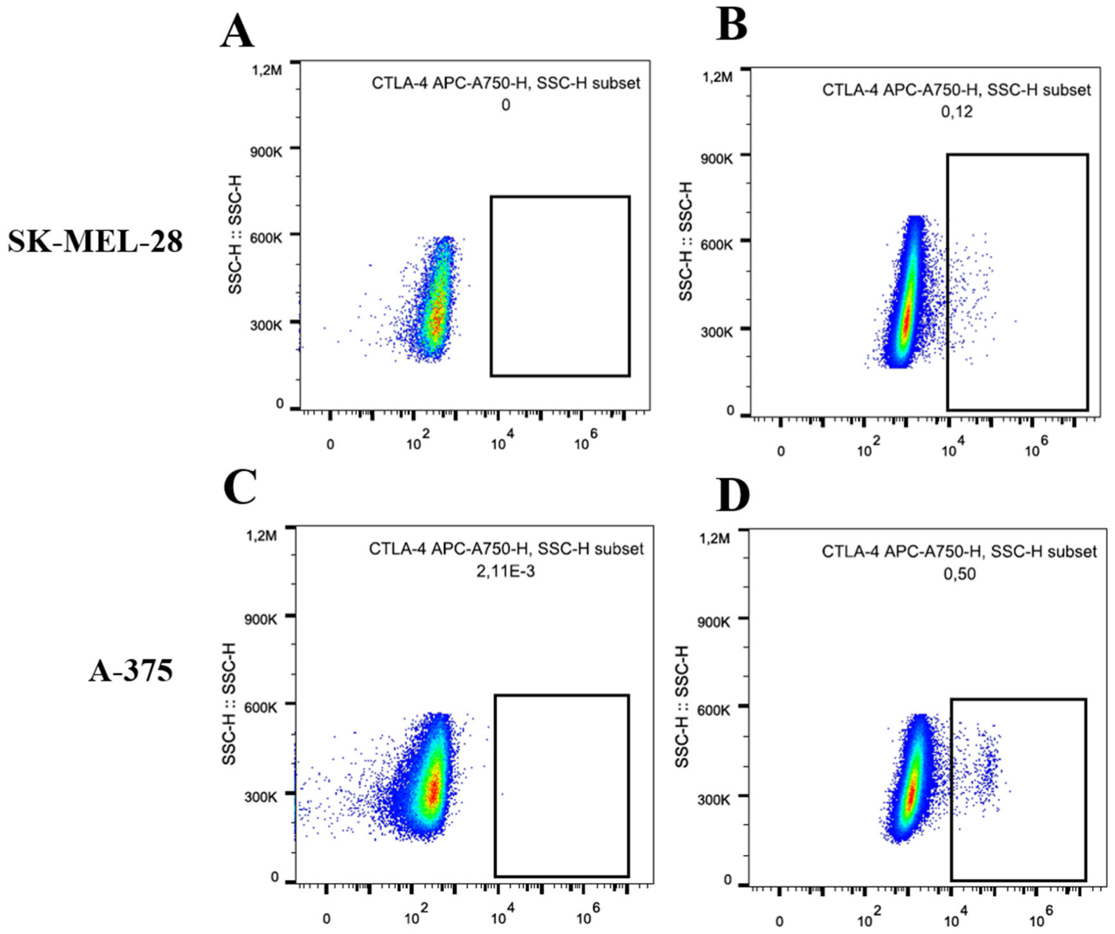

Figure S4. Analysis of *CTLA-4* expression in SK-MEL-28 (A, B) and A-375 (C, D) melanoma cell lines. Cells were analyzed for anti-CTLA-4 antibody staining on the cell membrane (A, C) and in the cytoplasm (B, D). Cells staining positive for anti-CTLA-4 antibody are represented in the rectangular area. CTLA-4 protein was identified in the cytoplasm of SK-MEL-28 and A-375 cells, but not in the cell membranes.
